# *Just in case*: undergraduate students identifying and mitigating barriers to their sexual and reproductive health needs

**DOI:** 10.1186/s12905-023-02854-7

**Published:** 2024-02-06

**Authors:** Rachel Olson, Jonathan Lehman, Angie Mejia, Rachael Ojeikhodion, Kristin Osiecki, Emily Kathambi, Silas Swarnakanth Kati, Anita Randolph

**Affiliations:** 1 Center for Learning Innovation, University of Minnesota Rochester, Rochester, MN USA; 2 Community Engagement and Education (CEEd) Hub, Masonic Institute for the Developing Brain (MIDB), University of Minnesota Twin Cities, Minneapolis, MN USA; 3 Center for Health Equity, Minnesota Department of Health, Minneapolis, MN USA; 4 University of Minnesota Twin Cities, Minneapolis, MN USA; 5 Department of Pediatrics, University of Minnesota Twin Cities, Minneapolis, MN USA

**Keywords:** Sexual and reproductive health (SRH), Undergraduate students, Sexual health education, Sexual health promotion, College health

## Abstract

**Background:**

Many U.S. colleges and universities offer access to a healthcare center that provides sexual and reproductive health (SRH) resources, services, and products. The importance of health centers in college and university settings in reducing sexual health disparities in student populations cannot be stressed enough. This article evaluates a student-led, mutual-aid, grassroots health promotion strategy for students with limited access to healthcare services, supplies, and tools via an anonymous and discrete distribution of SRH resources without charge.

**Methods:**

In partnership with faculty, undergraduate students worked to address their school’s unmet SRH needs by increasing on-campus access to comprehensive, evidence-based, and sex-positive resources. Referred to as *Just in Case*, this student-led, grassroots health promotion program provided students with supply kits containing contraceptives, sexual health wellness products, basic hygiene supplies, and education materials. Students were surveyed in a pre- *(n = 95)* post- *(n = 73)* pilot study to identify contraception acquisition barriers, discern perceptions of on-campus SRH resources, and elucidate trends in this program’s use and impact. Chi-square tests of independence were used to compare survey group responses, and association rule mining was employed in tandem to identify SRH items that students requested.

**Results:**

Students identified cost and privacy as significant barriers to acquiring sexual health products on campus. Of the 182 *Just in Case* supply kits requested by students during the 2022–2023 academic year, condoms were requested most frequently in 75% of fulfilled kits, while emergency contraception and pregnancy tests were asked most often in 61% of kits. 50% of students reported access to contraceptives on campus before this program’s implementation, growing to 75% (*p* < 0.001) 1 year later post-implementation. Similar jumps were observed for reported access to sexual health education (30 to 73%, *p* < 0.001) and services (36 to 73%, *p* < 0.001).

**Conclusion:**

A student-led SRH supply and resource delivery strategy may immediately reduce SRH inequities and decrease barriers to contraceptive use for students with limited access to on-site SRH product availability.

## Background

Sexual health disparities—such as sexually transmitted infections (STIs) [1], unintended pregnancies [2], and relationship-based [3, 4] and sexual violence [5, 6]—significantly impact U.S. post-secondary students’ educational trajectories and outcomes [7, 8]. For instance, risky sexual health practices [9], inconsistent or incorrect use of contraceptives [10], lack of access to sexual and reproductive health (SRH) preventive services [11, 12], resources and information [13], and gaps in sexual health knowledge due to differential access to comprehensive and scientifically-based sexual health education in K-12 contexts [14, 15], among others, have been connected to unplanned pregnancies, which can impact timely degree completion [16–18]. As more than 2/3 of students in U.S. colleges and universities are sexually active [1, 19], it is crucial to focus on and understand the impact of the health service provision context in place at their institutional homes [13].

Studies have stressed the importance of health centers in college and university settings in reducing sexual health disparities in student populations [20]. Students are more likely to visit a university health care center for their sexual health and family planning needs than other settings [11]. In addition, access to on-campus health services has been associated with students’ increased sexual health preventive behaviors such as STI testing [13, 21] and birth control use [9, 11]. A survey of 2- and 4-year post-secondary colleges in Minnesota found that sexually active students whose institutions provided access to on-campus sexual health services were less likely to report engaging in unsafe sex behaviors than those whose institutions did not have health centers [9]. Institutional factors, such as the size of the college, have been found to impact the use of barrier methods during sex, with students attending larger college campuses more likely to consistently use barrier methods during anal or vaginal sex compared to those enrolled in smaller campuses [22].

Research indicates that college students see their institutional home as responsible for providing sexual health knowledge, resources, and services [23] to the student body. This perspective of responsibility for service provision also includes students’ desires for their university health services to address issues of sexual and reproductive health in a supportive and affirming way [24]. However, universities and colleges vary in how they provide students with sexual health care services and resources. A nationally representative survey of colleges and universities in the U.S. estimates that approximately 70% had a healthcare center that provided sexual and reproductive health resources and services to their student body [25].

Students at colleges and universities lacking access to student-based healthcare services are at a disadvantage. Brindis and Reyes [26] reflect how students without access to campus-based health services often find themselves “visit[ing] emergency rooms for routine medical care…” [26], which puts them in an economically precarious situation that could threaten their educational outcomes**.** Taken together, we can see how students attending U.S. post-secondary settings without health care services bear a higher burden of sexual and reproductive health disparities than those enrolled in settings with services and resources at hand. As a response, universities and colleges with no campus-based health centers often attempt to meet this gap by referring students to outside providers [26, 27], outsourcing services [28], providing access to vending machines with health and wellness supplies [29], partnering with community-based organizations to provide health promotion and education [30, 31], and collaborating with health department settings to provide condoms and other supplies [32], among others. However, these responses and alternative avenues to health service provision are insufficient to address the apparent health disparity affecting populations enrolled in post-secondary settings without on-site student-based health care services.

This article presents the findings of a student-led, grassroots health promotion strategy at a health sciences undergraduate campus. Named *Just in Case,* this strategy is part of a larger grassroots and mutual-aid initiative between faculty and undergraduate students working together to address the sexual and reproductive health needs of undergraduates attending post-secondary settings with limited access to SRH resources by providing and increasing access to comprehensive, evidence-based, and sex-positive resources, and tools. This article evaluates the impetus, design, and use of this anonymous and discreet sexual and reproductive health supplies distribution strategy designed by undergraduate students and faculty supporters.

## Methods

The Health Sciences Institute (HSI) is an undergraduate health and medical sciences campus in the U.S. Midwest. At the time of writing, the population consisted of roughly 650 students**.** The student body encompasses various college-aged identity subgroups where 42% self-identify as non-white [33], 67% are from underrepresented groups, including low-income, first-generation, and those students identifying as racial/ethnic minorities [34], and approximately 80% are people self-identifying as women of reproductive age [33] (Table 1). The students at this campus have free access to a family medicine clinic [34, 35] that provides care to patients of all ages, contingent on paying a student services fee each semester [34, 35]. This clinic is available to the students and the general public, Monday through Friday between 8 am and 5 pm, except for an hour between noon and one [34, 35]. This clinic does not offer an embedded pharmacy or sell health supplies.

**Table 1:** Population-level and survey-level demographic characteristics

Demographics	HSI *(n = 646)*	Pre-Survey *(n = 95)*	Post-Survey *(n = 73)*
***Gender***	*–*	*𝜒*^*2*^*(1) = 0.059* *p = 0.808*	*𝜒*^*2*^*(1) = 1.838* *p = 0.175*
Female-identifying	79.4%	77.9%	83.6%
Male-identifying	20.6%	18.9%	13.7%
Non-binary identifying^a^	*NA*	3.2%	2.7%
***Age Group***	*–*	*𝜒*^*2*^*(1) = 0.014* *p = 0.906*	*𝜒*^*2*^*(1) = 0.122* *p = 0.727*
24 and under	95%	94.7%	95.9%
25 and over	5%	5.3%	4.1%
***Race***	*–*	*𝜒*^*2*^*(4) = 4.591* *p = 0.332*	*𝜒*^*2*^*(4) = 6.326* *p = 0.176*
American-Indian or Native Alaskan^b^	0%	1.1%	0%
Asian or Pacific Islander^c^	15%	11.6%	12.3%
Black or African American	12%	9.5%	13.7%
Hispanic or Latino	9%	8.4%	1.4%
White	58%	60.0%	67.1%
Two or more races	3%	7.4%	0%
Unknown/ Other^d^	2%	2.1%	5.5%

Demographic characteristics are included at the population level (campus is referred to as HSI) and at the group level (Pre-Survey and Post-Survey). Population-level demographics were obtained from the National Center for Education Statistics’ Fall 2021 enrollment data for HSI [33]. Chi-square goodness of fit tests were used to assess each survey group’s population representativeness; 𝜒^2^ and *p*-values are included for each demographic variable. No significant differences (*p* ≤ 0.05) were found across Gender, Age Group, or Race in either survey, supporting these groups as representative samples of the HSI undergraduate population

^a^Gender was reported as a binary in HSI’s enrollment data, while the surveys included non-binary options

^b^While ‘American-Indian or Native Alaskan’ was not included in the Chi-square test due to its 0% expected value, group proportions were still included in the table

^c^‘Asian’ (15%) and ‘Pacific Islander’ (0%) were reported separately in HSI enrollment data but were grouped together as a survey option

^d^The ‘Unknown/ Other’ grouping included respondents who selected ‘Other’ or did not report race

**Fig. 1:**
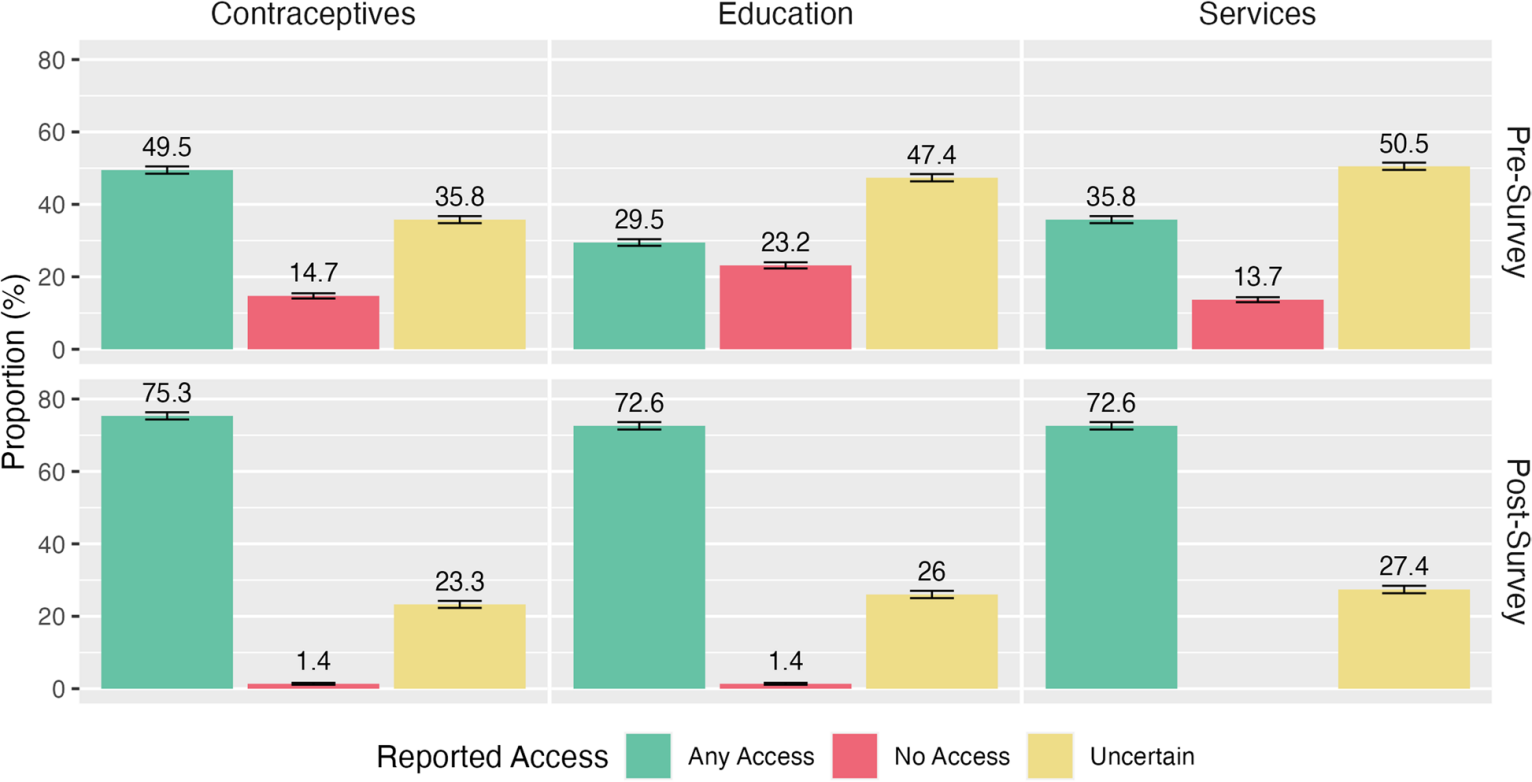
Pre- and post-survey responses on perceptions of on-campus access to contraceptives, SRH education, and services. *Students were asked to rate “How does HSI provide access to..”* (1) *“on-site resources of contraceptives that do not require a prescription (ex. condoms, spermicides, etc.)?”,* (2) *“sexual health education?” and* (3) *“sexual health services?”. Responses are presented as proportions and grouped per survey question, with 95% confidence intervals*

*Just in Case* began as a student-led, mutual-aid initiative in direct response to students reporting inadequate access to sexual health services, including education and products at HSI (Fig. 1). The program was designed to provide students with on-campus contraceptive and reproductive health resources by delivering SRH items directly to students through trusted student peers. The items available for request included condoms, emergency contraception (levonorgestrel), pregnancy tests, and essential hygiene products, including tampons, pads, razors, toothbrushes, and toothpaste (Table 2).

**Table 2:** Distribution of requested items during the *Just In Case* initiative

Requestable Item	Proportion of Requests *(n = 182)*
***Sexual Health***	***84.1%***
Condoms	74.7%
Dental Dams	21.4%
Lubricant	54.4%
***Family Planning***	***86.8%***
Emergency Contraception	74.2%
Pregnancy Test	73.6%
***Menstrual Products***	***61.0%***
Menstrual Pads	29.7%
Midol	16.5%
Panty Liners	13.2%
Tampons	39.0%
***Personal Hygiene***	***22.5%***
Personal Wipes	14.3%
Razors	12.1%
Toothbrush/ Toothpaste Kit	8.2%

The proportion of total orders (*n* = 182) that included each item during the 2022–2023 academic year are listed, with composite item groupings listed in italicized bold

In this pilot study, participants completed surveys to understand student perceptions of on-campus contraceptive availability, access to sexual health education, services, and on-site resources that supply contraceptives, as well as identify any barriers to obtaining contraceptives at school and desired on-campus services. Students were surveyed twice, once before implementing the *Just in Case* initiative and once post-program establishment in a pre-post study design, with potentially overlapping but distinct survey sample groups (Table 1). Students were also surveyed during *Just in Case’s* implementation to elucidate trends in *Just in Case* use and address reported barriers to accessing family planning and sexual health services, tools, and resources.

### Pre- and post-intervention surveys

Participants were recruited from the entire undergraduate study body via a convenience sampling strategy. Study recruitment information was disseminated using campus-wide advertisements, including print and social media advertising, word-of-mouth, flyers posted in residence halls, bathroom stalls, and by student service staff during on-campus socio-academic events. A 12-question survey was designed and administered, capturing data on participant demographics, perceived accessibility and availability of on-campus SRH provisions, barriers in contraceptive acquisition, perceptions of campus as a safe and supportive environment, and desired SRH resources. The pre-survey *(n = 95)* was administered in April 2022, and the post-survey *(n = 73)* was conducted 1 year later in April 2023. Institutional Review Board (IRB) approval was sought and obtained before dissemination of the Qualtrics survey. The University of Minnesota Institutional Research Board approved the protocol for this research. All surveys were conducted online and presented participants with written informed consent forms. Clicking ‘continue’ on the survey indicated a participant’s agreement to participate.

### Just in case: a student-led health initiative

Undergraduate students (Ojeikhodion and peers), with the guidance of faculty (Mejia and Olson), clinicians (Randolph), and health equity practitioners (Osiecki), designed *Just in Case* to be a student-centered anonymous and discreet intervention. Online and in-person product request options were available to students wishing to receive a supply kit. Online requests were completed via a Google Form made readily accessible to the student body via a QR code. *Just in Case* was advertised through campus-wide advertisements, including print and social media advertising. Students could opt for a “standard” order containing a pre-packaged set of products or tailor the request to their particular needs; students could place multiple requests throughout the year. Optimization of product availability has taken place through the duration of the *Just in Case* initiative as trends in specific product requests emerged. The university’s Family Educational Rights and Privacy Act (FERPA) liaison was consulted by Mejia before initiation to ensure this student initiative, including product distribution and research activities, was FERPA compliant.

Initially, the undergraduate student founders performed activities to sustain the day-to-day operations of *Just in Case* product acquisition, inventory, monitoring of student requests, order fulfillment, and general staffing and management of referrals to outside health resources. Packaged requests of *Just in Case* were made available for student pickup directly from the on-campus space designated for *Just in Case* operations. Shortly after the inauguration of this student-driven sexual health initiative, student volunteers and student engagement groups from a required community-engaged learning course supported the mission of *Just in Case.* All individuals supporting *Just In Case’s* activities received Health Insurance Portability and Accountability Act (HIPAA) and Research Ethics for Human Subjects training. General program costs, including *Just In Case* products and part-time student coordinator wages, were funded by institutionally managed grants, faculty research and development funds, and community contributions.

### Data analysis

To elucidate trends in product demand, we summarized the total requests for *Just in Case* kits from Fall 2022 and Spring 2023, with proportions of requestable products outlined in Table 2. We employed association rule mining to identify associations between the types of sexual and reproductive health products requested in tandem by students (Table 3).

**Table 3:** Association rule mining of *Just In Case* order requests

Antecedent	Consequent	Count	Support	Confidence	Lift
Dental Dams	Condoms	37	20.33%	**94.87%**	1.270
Emergency Contraception, Lubricant		65	35.71%	83.33%	1.115
Emergency Contraception, Pregnancy Test		92	**50.55%**	82.88%	1.109
Lubricant		83	45.60%	83.84%	1.122
Lubricant, Pregnancy Test		71	39.01%	83.53%	1.118
Lubricant, Tampons		43	23.63%	81.13%	1.086
Condoms, Pregnancy Test	Emergency Contraception	92	**50.55%**	86.79%	1.170
Condoms, Tampons		43	23.63%	81.13%	1.094
Lubricant, Pregnancy Test		72	39.56%	84.71%	1.142
Lubricant, Tampons		44	24.18%	83.02%	1.119
Menstrual Pads, Pregnancy Test		40	21.98%	85.11%	1.147
Pregnancy Test		111	**60.99%**	82.84%	1.117
Pregnancy Test, Tampons		50	27.47%	84.75%	1.142
Condoms, Tampons	Lubricant	43	23.63%	81.13%	1.492
Emergency Contraception, Tampons		44	24.18%	80.00%	1.471
Pregnancy Test, Tampons		48	26.37%	81.36%	1.496
Condoms, Emergency Contraception	Pregnancy Test	92	**50.55%**	88.46%	1.201
Condoms, Lubricant		71	39.01%	85.54%	1.162
Condoms, Menstrual Pads		37	20.33%	**90.24%**	1.226
Condoms, Tampons		46	25.27%	86.79%	1.179
Emergency Contraception		111	**60.99%**	82.22%	1.117
Emergency Contraception, Lubricant		72	39.56%	**92.31%**	1.254
Emergency Contraception, Menstrual Pads		40	21.98%	**95.24%**	1.294
Emergency Contraception, Tampons		50	27.47%	**90.91%**	1.235
Lubricant		85	46.70%	85.86%	1.166
Lubricant, Tampons		48	26.37%	90.57%	1.230
Menstrual Pads		47	25.82%	87.04%	1.182
Tampons		59	32.42%	83.10%	1.129

Thresholds for support and confidence were set at 20 and 80%, respectively. Values of support > 50% were bolded, indicating an itemset rule appeared in most order requests; values of confidence > 90% were also bolded, indicating a very high likelihood that if an antecedent was requested, so too would the corresponding consequent

Association rules analysis, used in epidemiology and bioinformatics research [36, 37], identifies patterns of items that co-occur in a dataset. Itemset rules, expressed as “*if A, then B*,” establish the likelihood of *B* (a consequent item) occurring, given the selection of *A* (an antecedent set). This conditional probability is also known as confidence. The strength of itemset rules can also be measured by support or the proportion of total orders in which an itemset (*A* & *B*) is found. A third measure, lift, is defined by the ratio between an itemset’s observed and expected support, given that *A* and *B* are independent. Values of lift greater than 1 suggest *A* and *B* occur together more frequently than by chance, pointing towards an association. This information provides greater insight into bundling purchases for items instead of focusing on the request history of each item individually. Given the limited sample size of requests *(n = 182)*, minimum thresholds (20% support, 80% confidence) were set for itemset rule generation. This allowed only the most prevalent and reliable itemsets to emerge, minimizing the likelihood of spurious findings.

To assess the impact of *Just in Case* on student perceptions of on-campus SRH resources, pre- and post-survey group responses were compared using Chi-square tests of independence. The representativeness of each sample group was assessed against the broader HSI undergraduate population using Chi-square goodness of fit tests on measures of gender, age group, and race.

All statistical analyses were conducted in R version 4.3.1 [38], using RStudio [39] and the tidyverse [40], arules [41], and eulerr [42] packages for table and figure generation.

## Results

### Preliminary survey findings

**Fig. 2:**
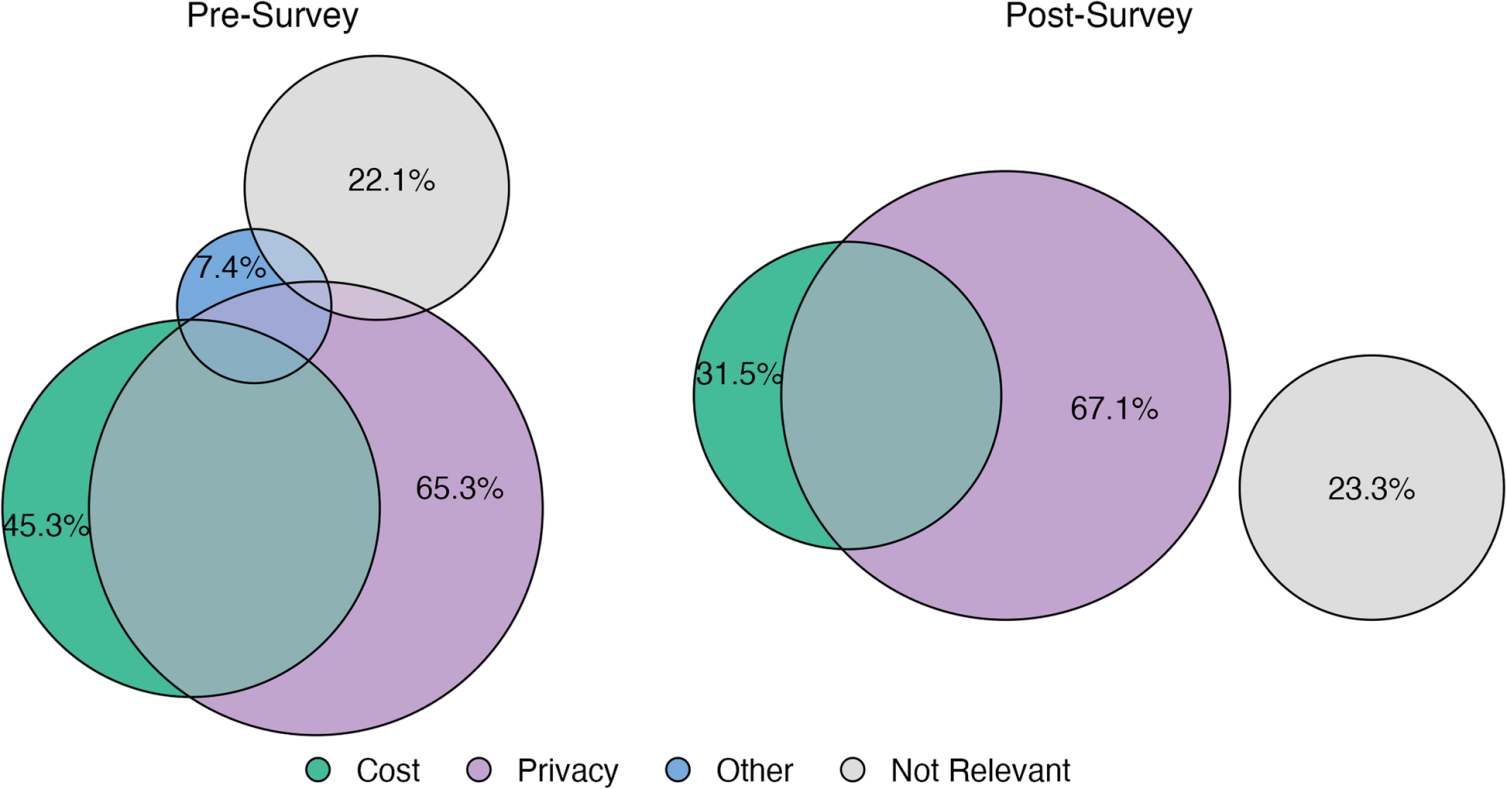
Pre- and post-survey comparison of perceived barriers to acquiring contraceptives. *The Euler diagrams show the barrier(s) reported by students answering the question: “What would stop you from getting contraceptives at school?”. Respondents were instructed to select all that apply from the options: “Cost,” “Privacy,” “Other,” and “Not Relevant.” Percentages represent the proportion of each survey group that selected a given barrier. Overlapping circles indicate the relative proportion of students who selected multiple barriers. No respondents selected “Other” in the post-survey group*

Preliminary data from an ongoing campus needs assessment revealed resource gaps impacting HSI students’ sexual and reproductive health practices. A survey of 95 students revealed that 51% were uncertain whether sexual health services were available on campus, while 14% reported no access to SRH services (Fig. 1). When queried about access to on-campus resources for acquiring contraceptives, a combined 50% of the respondents indicated uncertain or no access (Fig. 1). 65% of students included privacy concerns as a significant barrier to obtaining contraceptives on campus; 45% of respondents reported cost as a barrier (Fig. 2). While these preliminary survey results were underpowered, trends in undergraduate students’ knowledge of SRH and attitudes toward health behaviors in college support these findings. For instance, even when universities provide comprehensive health resources and services, undergraduates do not maximize the use of campus health services for their sexual and reproductive health needs [13].

### Just in case initiative

Over the course of the 2022–2023 academic year, 182 requests of *Just in Case* were fulfilled; of these requests, at least 100 came from first-time requesters. The highest items in demand included condoms, emergency contraception, pregnancy tests, lubricants, and tampons (Table 2). Association rule mining revealed that the groupings *“pregnancy test and emergency contraception”* and *“condoms, pregnancy test, and emergency contraception”* were the itemsets requested most frequently amongst students, appearing in 61 and 51% of orders, respectively (Table 3). Additionally, it was found that students requesting dental dams would also request condoms 95% of the time (Table 3). These trends were used to inform and optimize product availability for *Just in Case*.

A subgroup of students *(n = 49)* who received at least one kit completed an optional survey to understand student behaviors to advise future programming and demand for *Just in Case*. Of these students, 37% were sexually active at least once in the last week, 63% reported sexual activity in the previous month, and only 15% reported no sexual activity in the last 6 months, suggesting that sexually active individuals were the primary users of *Just in Case*. When queried on motivations for recent STI testing, 12% of students cited the educational materials included in kits as a motivator, demonstrating *Just in Case’s* ability to support informed SRH decision-making.

### Just in Case’s impact: pre- and post-analyses

*The Just in Case* initiative appears to have increased students’ perception of contraception availability on campus. When surveyed about on-campus contraceptive availability, a statistically significant difference was found between pre- and post-survey respondents using a Chi-square test of independence (𝜒^2^ (2) = 15.291, *p* < 0.001). 50% of respondents in the pre-survey reported on-campus contraceptive availability, compared to 75% of respondents reporting this post-survey (Fig. 1).

This student-led, anonymous, and discreet delivery strategy and method also impacted students’ perception of available sexual health education on campus. We asked students how HSI provides access to sexual health education. Using a Chi-square test of independence, we found a statistically significant difference (𝜒^2^ (2) = 35.175, *p* < 0.001) between the pre- and post-surveys for students’ reported level of access to sexual health education at this campus. Reports of education access rose from 30 to 73%, with drops in “No Access” and “Uncertain” responses (Fig. 1).

An increased perception of access to sexual health services on campus was also found (Fig. 1). The pre-and post-survey asked how HSI provides access to sexual health services. Using a Chi-square test of independence, a statistically significant difference (𝜒^2^ (2) = 26.248, *p* < 0.001) was found between the pre-and post-surveys for students’ reported level of access to sexual health services on this campus. Reports of access to sexual health services rose from 36 to 73%, with no post-survey respondents responding with “No Access,” compared to 14% who did in the pre-survey.

When students were asked about what stops them from acquiring contraceptives on campus, cost and privacy were reported as significant barriers (Fig. 2). No significant differences were found in the reported barriers between pre- and post-survey groups. However, it is worth noting that among the subgroup of *Just in Case* users who completed the optional kit survey *(n = 49)*, 69% identified cost as a barrier, significantly exceeding the proportions found in the pre-survey (𝜒^2^ (1) = 6.623, *p* = 0.010) and post-survey (𝜒^2^ (1) = 15.414, *p* < 0.001) groups (Fig. 2). This finding suggests that the primary beneficiaries of *Just in Case* are students who lack the financial stability to acquire contraceptives, affirming the initiative’s objective of addressing the unmet sexual and reproductive health needs of undergraduate students.

## Discussion

While college-aged youth’s access to sexual health resources and promotion can increase contraceptive use [14, 32] and decrease unsafe sex behaviors and practices [21, 43], sexual and reproductive healthcare disparities persist among groups of U.S. undergraduate students, especially those students attending institutions of higher education with limited access to student-based clinics and resources [1, 9, 25]. Furthermore, the burden of sexual health disparities in U.S. youth varies across identities [1, 4, 12, 27, 44, 45] and social determinants of health [1, 11, 27, 46], suggesting that access to SRH services, knowledge, and resources for U.S. students is a matter of increased urgency. This article highlighted preliminary findings of the effectiveness of a student-led, sexual, and reproductive health wellness delivery strategy among U.S. undergraduate students with limited access to SRH resources, services, and knowledge.

*Just in Case* may have increased students’ perception of contraception availability on HSI. Forty-two percent of respondents in the pre-survey reported on-campus contraceptive availability, compared to 71% of respondents reporting this post-survey. We suggest that the availability of *Just in Case* may have influenced this significant change. Previous studies found that students often needed to be aware of the resources and services available at their campuses [13, 21]. We should also note *Just in Case’s* impact on students’ perception of access to health services on campus. *Just in Case’s* approach to student recruitment and engagement—community-embedded and peer-to-peer direct messages about *Just in Case*—translated to campus availability to the students who used it.

This intervention may have also shaped students’ perception of available sexual health education on campus. There was a significant change between pre- and post-survey responses on the level of access to sexual health education. For instance, previous scholarship shows that limited sexual and reproductive health knowledge shapes college students’ use of available health services for SRH needs [11, 47, 48]. While *Just in Case’s* availability might have played a role in this drop, other factors may have also contributed. At the time of implementation, student leaders (Ojeikhodion and others) coordinated activities around implementing and delivering *Just in Case* and also organized student education events, workshops, presentations, and fairs in collaboration with Planned Parenthood and other reproductive justice organizations. Further, faculty members (Osiecki, Oslon, Randolph, and Mejia) designed, implemented and collaborated with students and *Just in Case’s* student leaders to develop community-engaged learning opportunities in and out of the classroom using Reproductive Justice-inspired [49] and participatory-centered educational activities geared to the campus community. The presence of these resources and events, in tandem with the availability of *Just in Case,* shaped students’ perspectives on the on-campus availability of SRH knowledge and resources.

Finally, the availability of *Just in Case* increased students’ perception of access to over-the-counter emergency contraceptives on campus, with a 28% increase in post-survey respondents who indicated “Easy Access.” There was also a change in those participants who responded to having “Minimum Access” and “No Access.” The availability of products in *Just in Case* and the connected health promotion and education activities comprising this larger initiative likely shaped this change in response. This finding supports previous research studies showing that college and university students prefer access to contraception and sexual health resources via a comfortable environment [43, 50] and discreet ways [20, 21, 51]. When implementing a mail-order contraceptive delivery program for college students, Butler and colleagues [52–54] found that receiving condoms and other sexual health aids via this modality allowed more discretion than a traditional campus health center setup, which in turn increased students’ ease when ordering sexual health supplies while decreasing high-risk behaviors via increased condom usage. Fluctuation in demand is expected to shift as *Just in Case* expands offerings based on student needs. Utilizing predictive analytics provides an opportunity to forecast future demand based on ongoing data collection with if/then operators.

### Implications for higher education settings with limited SRH service provision

Since university-provided student health services are increasingly supported by student fees and less institutional budget allocations [55], our findings apply to high-resourced institutional settings and those with limited resources and access. To increase comfort, institutional sites with no on-campus health centers should still focus on further leveraging ideas and strategies to increase regular STI/HIV testing, de-stigmatize seeking resources and treatment options for SRH concerns, and empower students to be more agentic in their health care behaviors with the resources already on-hand. Engagement in positive SRH behaviors is possible even in low-resource settings. For instance, strategies used by activists, health advocates, and practitioners when implementing peer-based promotion of contraceptives [56] in the Global South exemplify success despite resource limitations. *Just in Case’s* design and engagement activities were inspired by advocacy strategies used in these sites when reaching out to youth populations. Some of these novel approaches include the use of “community-embedded” [57] health promotion models that use participatory action research methods to educate, such as using documentaries based on sexual health norms with local community participants as the storytellers and educators [58]. Also, *Just in Case’s* flexible approach as a “pop-up” sexual health resource and knowledge, as well as a source of sexual health supply distribution [59], has been shaped by the lessons disseminated in recent papers highlighting the use of mobile health education consultations [60, 61]. Finally, in collaboration with Mejia and Osiecki, student leaders implementing *Just in Case* and other reproductive justice-centered activities have used participatory theater approaches similar to those used in youth-centered educational projects in parts of Latin America [57].

Using innovative self-directed testing for STIs might be another answer for low-resourced campus settings to increase SRH preventive behaviors in college-aged youth. For example, self-testing has been found efficient in undergraduate populations practicing riskier sex [62]. Such “at-home” approaches have been successful in populations located in low-resource contexts, domestically [63], and in the global South [64]. We see great promise in using these novel types of testing in tandem with solid partnerships with local and federally funded nonprofit health centers, including Title X Family Planning clinics.

### Implications for educators

Under the guidance of Olson and Mejia, undergraduate students identified, wrote, and submitted five grants over three semesters to support *Just in Case*, with one additional grant awaiting approval. The student grant-writers earned college credit as this experience was embedded into their coursework. Grant writing is a learned life-long skill that can transition from a daunting task to a successful experience through mentorship, scaffolded assignments, and constructive feedback [65–67]. While this manuscript focuses on *Just in Case’s* design, implementation, and success, it is worthwhile to note the rich learning opportunities for the students working to implement this intervention while enrolled in a community-engaged learning course. Briefly, the faculty worked with the students through grant identification, broke the components of the grants into attainable pieces and assignments, and assisted with the submission process. This experience fostered students’ problem-solving, scientific literacy, and communication skills [68, 69].

### Limitations

Although this pilot study highlights new understandings of undergraduates’ use of a sexual and reproductive health supplies distribution program, there are several limitations to note. For example, the survey did not measure the introduction of *Planned Parenthood* student advocates and activities in the Fall of 2022 and Spring of 2023. Our population comprises undergraduate students majoring in health and medical sciences, with most of them training to enter careers in healthcare. Students enrolled in majors related to health and medicine might be more likely to initiate SRH preventive behaviors than those selecting other majors [48]. Furthermore, our survey group sample sizes were smaller than desired, with each sample group representing approximately 10–20% of the total undergraduate population. Employing convenience sampling as our recruitment strategy may have also introduced self-selection bias to these samples. Together, these factors may limit the generalizability of our findings and the strength of our conclusions on undergraduate attitudes about SRH resources at HSI.

These limitations should be understood in the context that this was an undergraduate-led preliminary study to formally identify barriers to accessing SRH resources on campus, followed by a student-conceived and managed initiative to mitigate these barriers. It should also be noted that as was described in Table 1, the demographic compositions of our survey sample groups were not statistically different from the overall undergraduate population, lending support to their use as representative samples. In addition, our findings align with years of messages from the student body about a lack of SRH services and products. Before the pandemic, a group of students wrote a grant seeking to include SRH products in vending machines on campus; this action was quashed due to the pressing needs of the global pandemic and was subsequently forgotten about with time and student graduations. Finally, our implementation of *Just in Case* occurred during and throughout the social moment defined by the U.S. Supreme Court’s decision (Dobbs v. Jackson Women’s Health Organization) and now what activists call a post-Roe climate. Thus, findings concerning sexual and reproductive health access should be understood within those contexts.

## Conclusion

Sexual and reproductive healthcare disparities persist among groups of U.S. undergraduate students despite increased efforts at the policy level to provide healthcare coverage to U.S. individuals and at the institutional level to increase access to available services. The above findings highlight the importance of further research into intragroup health disparities beyond enrollment in the types of institutions and services provided. A future goal will be to continue a deeper evaluation and dissemination of replicable practices that individuals and groups in similar low-resourced contexts can quickly implement to increase existing health service use. Our ethnographic forays and connected qualitative research findings into the lives of our students indicate this population’s need to strengthen their sense of sexual citizenship in a current moment where rights related to one’s embodiment and sexuality are constantly under attack.

## Data Availability

The data and materials analyzed for this study (including supplementary work) are available from the corresponding author upon reasonable and on-time request.
